# Diversity within Italian Cheesemaking Brine-Associated Bacterial Communities Evidenced by Massive Parallel 16S rRNA Gene Tag Sequencing

**DOI:** 10.3389/fmicb.2017.02119

**Published:** 2017-11-03

**Authors:** Marilena Marino, Nadia Innocente, Michela Maifreni, Jérôme Mounier, José F. Cobo-Díaz, Emmanuel Coton, Lisa Carraro, Barbara Cardazzo

**Affiliations:** ^1^Dipartimento di Scienze Agroalimentari Ambientali e Animali, Università degli Studi di Udine, Udine, Italy; ^2^Laboratoire Universitaire de Biodiversité et Ecologie Microbienne, Institut Brestois Santé Agro Matière (IBSAM), École Supérieure d’Ingénieurs en Agroalimentaire de Bretagne Atlantique (ESIAB), Université de Brest, Plouzané, France; ^3^Dipartimento di Biomedicina Comparata e Alimentazione, Università degli Studi di Padova, Padova, Italy

**Keywords:** cheese brines, bacterial diversity, 16S rRNA gene amplicon sequencing, cheese type, salinity, *Staphylococcus aureus*, *Tetragenococcus halophilus*

## Abstract

This study explored the bacterial diversity of brines used for cheesemaking in Italy, as well as their physicochemical characteristics. In this context, 19 brines used to salt soft, semi-hard, and hard Italian cheeses were collected in 14 commercial cheese plants and analyzed using a culture-independent amplicon sequencing approach in order to describe their bacterial microbiota. Large NaCl concentration variations were observed among the selected brines, with hard cheese brines exhibiting the highest values. Acidity values showed a great variability too, probably in relation to the brine use prior to sampling. Despite their high salt content, brine microbial loads ranged from 2.11 to 6.51 log CFU/mL for the total mesophilic count. Microbial community profiling assessed by 16S rRNA gene sequencing showed that these ecosystems were dominated by *Firmicutes* and *Proteobacteria*, followed by *Actinobacteria* and *Bacteroidetes*. Cheese type and brine salinity seem to be the main parameters accountable for brine microbial diversity. On the contrary, brine pH, acidity and protein concentration, correlated to cheese brine age, did not have any selective effect on the microbiota composition. Nine major genera were present in all analyzed brines, indicating that they might compose the core microbiome of cheese brines. *Staphylococcus aureus* was occasionally detected in brines using selective culture media. Interestingly, bacterial genera associated with a functional and technological use were frequently detected. Indeed *Bifidobacteriaceae*, which might be valuable probiotic candidates, and specific microbial genera such as *Tetragenococcus, Corynebacterium* and non-pathogenic *Staphylococcus*, which can contribute to sensorial properties of ripened cheeses, were widespread within brines.

## Introduction

Salting represents a crucial step in the manufacture of most cheese varieties. Brine salting is the most commonly used method to increase cheese salt content, and it consists in soaking molded curd from few minutes to several days in a brine solution containing NaCl concentrations ranging from about 5–25%. Brine-salting is usually applied on completion of molding and pressing of cheese curd to final size/shape of cheese. The duration of salting process is from few hours to several days, depending on size and shape of cheese, NaCl content of brine and temperature, and required salt content in finished cheese (Guinee, 2007b). During this period, the difference in osmotic pressure between the cheese moisture and the brine drives the movement of NaCl molecules, as Na^+^ and Cl^-^, from the brine to the curd. Simultaneously, water and other compounds including soluble proteins, fats, sugars, lactate, and minerals are expelled from the curd, which makes the brine a nutrient-rich medium. Due to economic and environmental pollution issues, cheese brines are only rarely replaced, and more frequently only fresh salt is added regularly to replenish the lost salt. Moreover, using fresh brine can result in low-quality cheese due to the so-called “soft-rind defect,” so it is preferable not to replace it completely but regenerate it using chemical or thermal ways (Guinee, 2007c). Since brines are used over long time periods (up to several months) and for several cheesemaking batches, microbial counts originating from salt, water curd, and equipment usually increase significantly over time (Schirmer et al., 2014). These microorganisms come in contact with the cheese rind during immersion in the brine, and it is conceivable that their metabolism can modify in some way the product characteristics and its organoleptic profile. It has been shown that the cheese surface of traditionally aged cheeses harbors biofilm composed of a large microbial diversity originating from raw milk, starter, and adjunct cultures, as well as from brine and dairy environment (Mounier et al., 2006a). Moreover, cheese brines, except for the case of white pickled cheese such as Feta cheese, are often considered as a possible contamination source of the curd. Indeed, numerous studies have focused on the effect of various conventional and unconventional antimicrobial treatments, such as pasteurization, microfiltration, UV-treatment, and ozonation, on brine microbiota (Merin et al., 1983; Saboya and Maubois, 2000; Bintsis et al., 2000; Marino et al., 2015). Microbial composition of cheese brines is poorly documented, and almost exclusively focused on the possible presence of pathogenic microorganisms (Larson et al., 1999; Osaili et al., 2014). These studies reported that brine microbiota is mainly composed of halotolerant microorganisms, including corynebacteria, microstaphylococci, yeasts, and molds; however, used brines can also harbor pathogenic bacteria such as *Listeria monocytogenes* (Larson et al., 1999; Wemmenhove et al., 2014). In the latter studies, culture-dependent methods were used, however, it is well known that, in environments characterized by high salinity and growth-inhibiting temperatures, as well as nutrient starvation, anoxia, and stressful pH, bacteria are able to enter a Viable But Non-Culturable (VBNC) state, rendering some of them undetectable by culture-based methods (Gin and Goh, 2013).

Over the last few years, high-throughput next generation sequencing (NGS), together with advanced molecular methods, has considerably improved the field of food microbiology. By avoiding the cultivation step and thus overcoming biases associated with this approach, it has become possible to achieve novel insights into the microbial diversity associated with food. Using amplicon sequencing, it is now possible to obtain, from a single sample, a large number of sequences that are clustered into groups defined as Operational Taxonomic Units (OTUs) and classified through comparisons against sequence databases to obtain an almost complete description of microbial communities, including both dominant and minor microbial populations. NGS has been successfully carried out to decipher the microbial consortia of dairy, meat and vegetable foods (Ercolini et al., 2012; Jackson et al., 2013; Coton et al., 2017). Although the role of the processing environment, including that of the brine, as a potential reservoir for bidirectional microbial transfer between cheese fermentations has been underlined (Bokulich and Mills, 2013), to our best knowledge, cheese brine microbiota has only been assessed once using an rRNA gene-based culture-independent high-throughput sequencing, and in a very limited way, since only one brine sample used for Caciocavallo cheesemaking was studied for bacterial and fungal community composition (Stellato et al., 2015). However, many cheese varieties exist differing in their cheesemaking and ripening practices, and it is likely that these have a major influence on brine microbiological characteristics.

In this study, commercial brines used for soft, semi-hard, and hard Italian cheeses were collected in different cheese plants and characterized for their physicochemical parameters and microbial viable counts. They were also analyzed through an amplicon sequencing approach in order to describe the bacterial microbiota inhabiting cheese brines.

## Materials and Methods

### Brine Samples

Nineteen commercial brine samples were obtained from 14 cheese plants located in North–East, Central, and Southern Italy. 500 mL of brines were sampled aseptically, cooled, and transported to the laboratory at +4°C before physicochemical and microbiological analyses. The selected brines were in use for at least 2 months but were not in contact with curd at the time of sampling. Information on cheese type and handling methods associated with the sampled brines are presented in **Table 1**.

**Table 1:** Physicochemical characteristics and microbial viable counts (log CFU/mL) of cheese brine samples.

Brine	NaCl	pH	Acidity	Proteins	Total	Lactic	Microsta	Yeasts	Molds	Coliforms	*E. coli*	*S. aureus*
	(g/100 g)		(°SH/50 mL)	(g/100 g)	mesophilic	acid	phylococci					
					count	bacteria						
B_S1	13.95^c^	4.80^bc^	10.07^a^	0.46^b^	5.61^a^	5.93^a^	3.85^c^	4.85^ab^	n.d.^b^	1.40^a^	n.d.^c^	n.d.^b^
B_S2	14.41^c^	4.98^bc^	8.07^b^	0.21^d^	6.51^a^	6.41^a^	4.76^b^	5.86^a^	1.60^a^	0.78^b^	n.d.^c^	n.d.^b^
B_S3	15.74^bc^	4.64^c^	5.75^c^	0.20^d^	4.40^b^	4.49^b^	5.66^ab^	3.11^bc^	n.d.^b^	1.95^a^	1.00^b^	n.d.^b^
B_S4	12.88^d^	4.99^bc^	4.18^d^	0.18^d^	4.71^b^	2.30^d^	4.07^c^	n.d.^e^	n.d.^b^	n.d.^b^	n.d.^c^	n.d.^b^
B_SH1	9.45^e^	5.05^b^	11.33^a^	0.30^c^	4.64^b^	5.90^a^	3.70^c^	3.95^b^	2.00^a^	2.18^a^	1.30^b^	n.d.^b^
B_SH2	7.45^f^	5.02^b^	11.93^a^	0.33^bc^	5.59^a^	5.64^a^	4.11^bc^	5.21^a^	n.d.^b^	2.56^a^	n.d.^c^	1.30^a^
B_SH3	12.17^d^	5.05^b^	10.40^a^	0.26^c^	4.15^b^	5.23^a^	4.04^c^	3.99^b^	n.d.^b^	n.d.^b^	n.d.^c^	0.48^b^
B_SH4	14.24^c^	5.00^b^	9.44^ab^	0.17^d^	5.61^a^	5.95^a^	4.72^b^	4.96^ab^	n.d.^b^	n.d.^b^	1.00^b^	1.00^a^
B_SH5	15.74^bc^	5.12^ab^	2.94^e^	0.08^e^	4.70^b^	5.74^a^	6.46^a^	4.40^b^	n.d.^b^	n.d.^b^	n.d.^c^	n.d.^b^
B_SH6	12.47^d^	5.05^b^	9.72^ab^	0.38^bc^	5.60^a^	5.76^a^	3.57^c^	5.72^a^	1.00^a^	2.00^a^	n.d.^c^	n.d.^b^
B_SH7	13.15^cd^	5.00^b^	9.22^ab^	0.29^c^	4.67^b^	4.18^b^	4.22^b^	3.61^bc^	n.d.^b^	2.20^a^	3.29^a^	n.d.^b^
B_SH8	15.46^bc^	5.20^ab^	7.72^b^	0.38^bc^	2.11^d^	2.16^d^	4.15^bc^	n.d.^e^	n.d.^b^	n.d.^b^	n.d.^c^	n.d.^b^
B_SH9	16.32^b^	5.03^b^	6.54^bc^	0.29^c^	3.92^c^	5.32^a^	3.32^c^	1.30^e^	n.d.^b^	n.d.^b^	n.d.^c^	n.d.^b^
B_SH10	14.23^c^	5.11^ab^	5.93^c^	0.36^bc^	3.46^c^	2.00^d^	5.48^ab^	3.11^bc^	n.d.^b^	n.d.^b^	n.d.^c^	n.d.^b^
B_H1	17.22^b^	5.57^a^	6.02^c^	0.60^a^	4.72^b^	3.59^c^	4.56^b^	2.63^c^	n.d.^b^	n.d.^b^	n.d.^c^	n.d.^b^
B_H2	17.61^b^	5.42^a^	2.83^e^	0.20^d^	3.95^c^	3.60^c^	4.36^b^	2.20^c^	n.d.^b^	n.d.^b^	n.d.^c^	n.d.^b^
B_H3	15.84^bc^	5.67^a^	5.05^cd^	0.12^e^	4.85^b^	2.30^d^	5.42^ab^	2.62^c^	n.d.^b^	n.d.^b^	n.d.^c^	n.d.^b^
B_H4	17.66^b^	5.32^a^	5.88^c^	0.46^b^	5.78^a^	3.49^c^	4.46^b^	1.60^e^	n.d.^b^	n.d.^b^	n.d.^c^	n.d.^b^
B_H5	19.57^a^	5.36^a^	5.32^cd^	0.34^bc^	6.21^a^	3.95^bc^	3.37^c^	3.20^bc^	n.d.^b^	n.d.^b^	n.d.^c^	n.d.^b^

*n.d. ≤ 10 CFU/mL (for yeasts and molds) or <1 CFU/mL (for coliforms, *E. coli* and *S. aureus*). The values represent means of duplicates; means followed by different letters in the same column are significantly different (*p* < 0.05) according to Tukey’s HSD test; for statistical analysis, values of <10 and <1 were treated as log (9) and log (0.9), respectively.*

### Physicochemical Analyses

Brines were analyzed for titratable acidity, protein, and sodium chloride content, as previously reported by Marino et al. (2015). pH was determined directly using a pH meter at controlled temperature (Hanna Instruments, mod. pH 301, Villafranca Padovana, Italy) (Biasutti et al., 2010). Temperature of brines was measured at the cheese plants using a handheld infrared thermometer with laser pointer (Maplin Electronics, United Kingdom) (Segat et al., 2016).

### Microbiological Analyses

For culture-based microbiological analyses, since cheese brines contain significant concentrations of salt, diluent and culture media supplemented with 10% w/v NaCl were used in a preliminary step, and microbial populations were compared with non-supplemented media. Because no statistically significant differences were found between viable counts (*p* > 0.05), the analyses were then performed in non-supplemented media. Brine samples were decimally diluted in Maximum Recovery Diluent (Oxoid, Milan, Italy) and examined for microbial populations using the spread-plate or the pour-plate method. By the spread-plate method, 1 mL of sample or its dilution was pipetted onto the agar surface and spread evenly using a sterile glass spreader, by the pour-plate method 0.1 mL of sample or its dilution was pipetted into a sterile plate, then the sterile medium was added and mixed well with the inoculum. Samples were analyzed for total mesophilic count on Gelatin Sugar-Free Agar (incubation at 30°C for 48 h), microstaphylococci on Mannitol Salt Agar (30°C for 48 h), lactic acid bacteria (LAB) on MRS agar pH 5.4 with 0.025% Delvocid (DSM, Heerlen, the Netherlands) (30°C for 48 h under anaerobic conditions), yeasts and molds on Oxytetracycline Glucose Yeast Extract Agar (25°C for 72 h), coliforms and *Escherichia coli* on ColiID medium (37°C for 24 h), and *Staphylococcus aureus* on Baird Parker RPF Agar (37°C for 48 h). All culture media were obtained from Oxoid (Milan, Italy), except for ColiID and Baird Parker RPF Agar (bioMérieux, Marcy l’Etoile, France).

### RNA Extraction and 16S rRNA Library Preparation

Fifty mL of brine aliquots were centrifuged at 4,000 rpm for 30 min at + 4°C and RNA was extracted from the cell pellet using the RNAeasy Mini kit (Qiagen, Hilden, Germany) according to the manufacturer’s instructions. RNA concentration and quality were measured using a UV-Vis spectrophotometer NanoDrop ND-1000 (Nanodrop Technologies, Wilmington, DE, United States) and by qPCR using 16S rRNA primers (Martin et al., 2002). One microgram of RNA for each sample was reverse transcribed to cDNA using the Superscript II kit (Invitrogen, Life Technologies, Monza, Italy). cDNA was amplified with primers targeting the 16S rRNA gene V3-V4 region as described in Carraro et al. (2011). The primer includes sequences required for Illumina platform sequencing, a barcode and sequences corresponding to the bacterial 16S rRNA gene. cDNA was diluted to 0.2 ng/μL and amplified in three 20-μL reactions per sample, each composed of 5 μL of diluted cDNA, 0.4 μM of each primer (PCR1_16S_For CTA CAC GAC GCT CTT CCG ATC TTC CTA CGG GAG GCA GCA GT; PCR1_16S_Rev **CAG ACG TGT GCT CTT CCG ATC** TGG ACT ACC AGG GTA TCT AAT CCT GTT; the adapters are highlighted in bold), 0.25 mM dNTPs, 1× Phusion HF buffer and 1 U Phusion high-fidelity DNA polymerase (New England BioLabs, Ipswich, MA, United States). PCR was performed in a 2720 thermal cycler (Applied Biosystems, Waltham, MA, United States) with 25 cycles at 95°C for 30 s, 60°C for 30 s and 72°C for 45 s and then a final extension for 7 min at 72°C. Products were purified using the AMPure purification kit (Agencourt, Beverly, MA, United States) and after beads purification, the targeted band was checked on a 1.8% agarose gel. Barcodes were introduced by a second PCR with platform-specific barcode-bearing primers. Each 50-μL PCR reaction contained 5 μL of PCR products, 0.2 μM of each primer (PCR2_For1 AAT GAT ACG GCG ACC ACC GAG ATC TAC ACT CTT TCC CTA CAC GAC GCT CTT CCG ATC T; PCR2_Rev1 CAA GCA GAA GAC GGC ATA CGA GAT xxxxxxx GT GAC TGG AGT TCA GAC GTG TGC TCT TCC GAT C; PCR2_For2 AAT GAT ACG GCG ACC ACC GA; PCR2_Rev2 CAA GCA GAA GAC GGC ATA CGA), 0.3 mM dNTP, 1x Phusion HF buffer and 1 U Phusion high-fidelity DNA polymerase. Ten cycles of the PCR profile listed above were performed. PCR products were purified using AMPure purification kit. After final purification, every sample was quantified using the Qubit 2.0 Fluorometer (Invitrogen, Life Technologies, Monza, Italy). The quality of amplicon libraries was tested by using an Agilent 2100 Bioanalyzer (Agilent Technologies, Palo Alto, CA, United States). Libraries were pooled and sequenced by BMR Genomics (Padova, Italy) using Miseq Illumina 2x300 version 3.

### Sequence Identification and Bioinformatics Analyses

For each sample, 200,000–250,000 raw reads with an average length of 476 bp were generated. Raw sequence data were split into samples according to their respective barcode, and low-quality reads were filtered using the CLC Genomics Workbench (Illumina Pipeline 1.5–1.7 and trim quality score 0.05). Miseq 300 PE sequencing of the described libraries generated data with overlapping pairs. To merge the overlapping (mismatch cost 2, minimum score 10, gap cost 3) pair into one sequence read data, CLC Genomics software was used. 16S rRNA sequence data was then processed using the software QIIME (Quantitative Insights Into Microbial Ecology) (Caporaso et al., 2010). The sequences were clustered into OTUs based on their sequence similarity at 0.97 using UCLUST against the chimera-checked 16SrRNA Greengenes database (Edgar, 2010). A representative sequence for each OTU was chosen for downstream analysis based on the most abundant sequence from each OTU and only OTUs represented by more than 5 reads were considered. PyNAST was used to align sequences. A tree was constructed with a set of sequences representative of each OTU using FastTree (Price et al., 2010). The sequence data file was deposited in the SRA database with accession number SRP109273. Diversity within a sample (α-diversity) and between samples (β-diversity) was assessed. The α-diversity metrics Chao1 (species richness), observed Species (count of unique OTUs found in the sample), Shannon and PD whole tree were estimated.

### Statistical Analyses

One-way analysis of variance was carried out on the mean values obtained from physicochemical and microbiological analyses to determine significant differences at *p* < 0.05. Differences between the means were assessed using the Tukey’s HSD *post hoc* test (Statistica v. 8, Statsoft Inc., Tulsa, OK, United States). β-diversity was used to generate principal coordinate analysis plots based on UniFrac and Bray-Curtis distances. UniFrac distances were calculated in two different ways, using only presence/absence information (unweighted) or taking into account the abundance of each bacterial lineage (weighted). Heatmaps were plotted using the R package heatmap with “euclidean” distance and “complete” method for clustering. The Calypso software tool was used for further statistical comparisons (Zakrzewski et al., 2017).

## Results

### Physicochemical and Microbiological Characteristics of Brines

In this study, 19 brines with different geographical origin and associated with different Italian cheese types, including soft, semi-hard, and hard cheeses, were studied. The brines were first studied for their management at dairy plants and their physicochemical characteristics, since it is expected that it may have some consequences on the composition of the microflora. Brine handling was automatic in six out of nineteen brines, while in other cases it was manual (Supplementary Table S1). Interestingly, in most hard cheeses salting operations were handled automatically, presumably to make salt penetration faster in such curds, which have to be ripened for very long periods.

Temperatures measured inside brine tanks at the cheese plants ranged from 8 to 16°C, and the highest temperatures were recorded in hard cheese brines. Hard cheese brines had also the highest values in salinity and pH (**Table 1**). These parameters showed both large variations. Indeed, NaCl concentrations ranged from 7.45 to 19.57 g/100 g, and pH values from 4.64 to 5.67. The lowest pH values were detected in brines for soft cheeses. Acidity ranges were quite wide (2.83-11.93°SH/50 mL) within all studied cheese varieties since this value is affected not only by the type of soaked cheese but also by the use duration and daily workload. Total protein ranged from 0.08 to 0.60 g/100 g, and a high variation within the cheese varieties was observed.

Overall, brine microbial loads were significant, despite high salt concentrations. Total mesophilic counts were highly variable, ranging from 2.11 and 6.51 log CFU/mL. Lactic acid bacteria were present at concentrations comprised between 2.00 and 6.41 log CFU/mL and likely originated from the curd. A few brines were contaminated by molds, whereas high viable counts of microstaphylococci and yeasts were quite common. Indeed, microstaphylococci and yeasts counts ranged from 3.32 to 6.46 log CFU/mL and 1.30 to 5.86 log CFU/mL, respectively. Coliforms were detected in seven out of 19 brines, with a maximum concentration of 2.56 log CFU/mL. *E. coli* and *S. aureus* were detected in four and three brines, respectively.

### Composition and Comparison of Brine Bacterial Communities

Bacterial communities of brine samples were assessed by 16S rRNA gene amplicon sequencing. Retrotranscribed RNAs were used as template for library construction as a better representative of the living microbiota. Overall, 678 OTUs were detected considering the communities from all samples, of which 489 were classified at the genus level and 66 at the species level. α-diversity metrics estimated after rarefaction demonstrated that the OTUs were distributed among samples from a minimum of 71 (brine B_SH10) to a maximum of 437 (B_H5), as reported in **Table 2**. Diversity indices (PD whole tree, Chao1, and Shannon) revealed a high diversity within all brine samples, with the highest values in samples B_H1, B_H2, and B_H5.

**Table 2:** Diversity indices, coverage, and number of reads obtained from the brines.

Brine	Reads	Observed OTUs	PD whole tree	Chao1	Shannon
B_S1	104,105	145	9.13	233.01	2.35
B_S2	118,258	231	12.25	369.88	3.45
B_S3	70,463	93	7.39	138.60	2.15
B_S4	81,981	209	11.49	290.05	2.36
B_SH1	105,197	134	8.40	205.92	2.20
B_SH2	82,886	178	10.84	288.41	1.86
B_SH3	81,123	204	10.79	274.69	3.88
B_SH4	89,714	172	10.75	247.21	2.51
B_SH5	81,475	125	7.94	177.30	1.33
B_SH6	53,489	185	11.46	260.86	3.06
B_SH7	74,380	245	13.46	377.48	3.21
B_SH8	108,209	133	8.35	236.06	2.54
B_SH9	79,524	277	15.98	392.14	2.30
B_SH10	134,390	71	6.62	129.04	0.83
B_H1	84,012	353	20.05	590.39	3.06
B_H2	89,445	379	21.78	455.27	4.25
B_H3	91,400	117	7.69	159.91	2.98
B_H4	86,367	127	8.69	174.71	2.02
B_H5	90,379	437	19.76	576.22	4.62

Brine bacterial communities were dominated by members of the *Firmicutes* and *Proteobacteria*, followed by *Actinobacteria* and *Bacteroidetes phyla*. The relative abundance of these four *phyla* was quite variable among the samples, ranging within very wide intervals. In one brine sample (B_H2), members of *Fusobacteria* were detected. Two candidate divisions, namely GN02 and TM7, were identified at low prevalence (<0.1%) in six and two brines, respectively. Even though the primers used to amplify cDNA targeted the V3-V4 region of 16S rRNA gene, members of *phylum Euryarcheota* were detected in seven brines (0.89% of the reads), used for one soft, four semi-hard and two hard cheeses, respectively.

**FIGURE 1:**
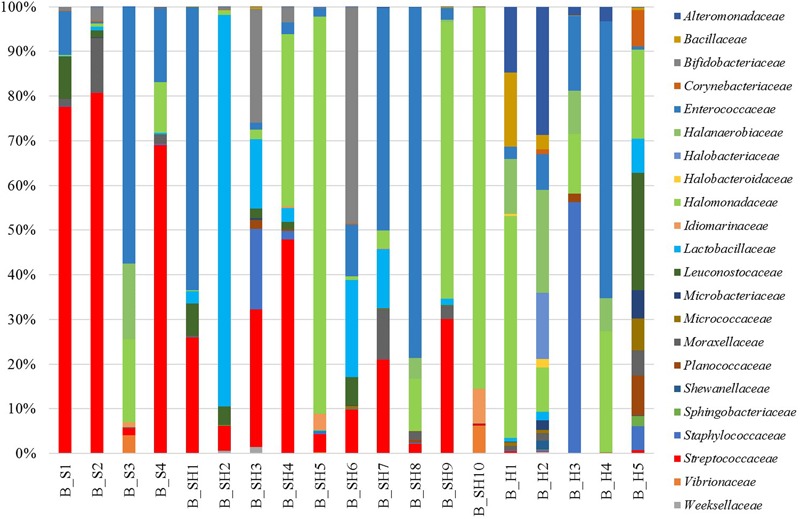
Relative abundance of OTUs at the family level. Only OTUs with relative abundance > 1% in at least one sample are shown.

Sixty-three families were detected, and 22 of them were present at abundance > 1% in at least one sample (**Figure 1**). The most abundant bacterial families were *Lactobacillaceae, Moraxellaceae, Halomonadaceae, Streptococcaceae, Micro-coccaceae, Pseudomonadaceae*, and *Staphylococcaceae* that were present in all tested brines. Many of the dominant families were widely distributed across the samples, but their abundance within each brine community was variable, somehow related to the type of cheese soaked.

**FIGURE 2:**
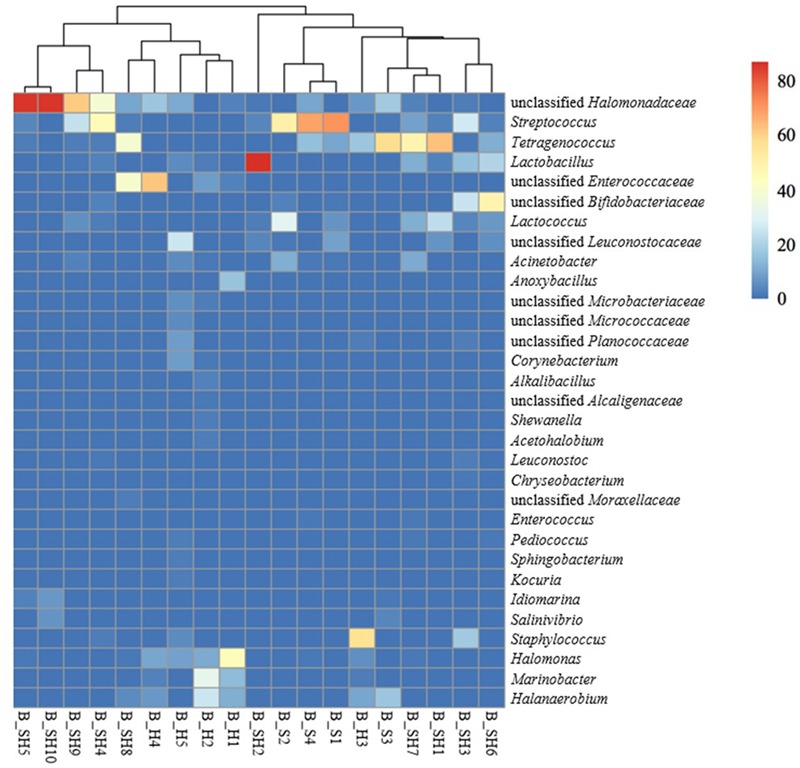
Heatmap showing the relative abundance of OTUs at the genus level. Only OTUs with relative abundance > 1% in at least one sample are shown. The color code indicates the range of relative abundance for a given genus.

Analyzing the microbial diversity to the deeper taxonomic assignment, a total of 99 different genera were identified, 22 of which were present at least at 1% in at least one brine sample (**Figure 2**). Nine genera (*Acinetobacter, Halomonas, Idiomarina, Lactobacillus, Lactococcus, Staphylococcus, Streptococcus, Tetragenococcus*, and *Pseudomonas*) composed the core microbiome of cheese brines, since they were found in all tested brines, even if their abundance varied depending on the brine sample. The *Tetragenococcus* genus was quite widespread in brines and its prevalence was even higher than 50% in two brines. *Staphylococcus* spp. were also quite common.

Fifty four OTUs were identified down to the species level (Supplementary Table S2). *S. aureus* was present in fourteen brines. Furthermore, other opportunistic pathogens were found in repeatedly used brines, as *Acinetobacter johnsonii*, which was detected in almost all samples. Confirmation of *S. aureus* and other pathogens’ presence and determination of their abundance using quantitative PCR might be performed to confirm our results. Moreover, *Propionibacterium acnes, Shewanella algae*, and coagulase-negative staphylococci, were frequently found. Several species with a technological role in dairy foods were detected in brines, such as *Corynebacterium variabile, Microbacterium maritypicum, Pediococcus acidilactici* and *Staphylococcus equorum*. Moreover, species with a probiotic potential were present, for example, various lactobacilli, *Leuconostoc mesenteroides* and *Tetragenococcus halophilus*.

The β-diversity among brine microbiota was estimated using Bray-Curtis distances and visualized using a Principal Coordinate Analysis (PCoA) while variance analysis of these distances was performed using a multivariate permutation Adonis test including cheese plant, cheese variety, curd handling, temperature, NaCl level, pH, acidity and protein content factors. At the OTU level, cheese type (*p* = 0.0007), cheese plant (*p* = 0.009) and NaCl range (*p* = 0.003) shaped β-diversity.

**FIGURE 3:**
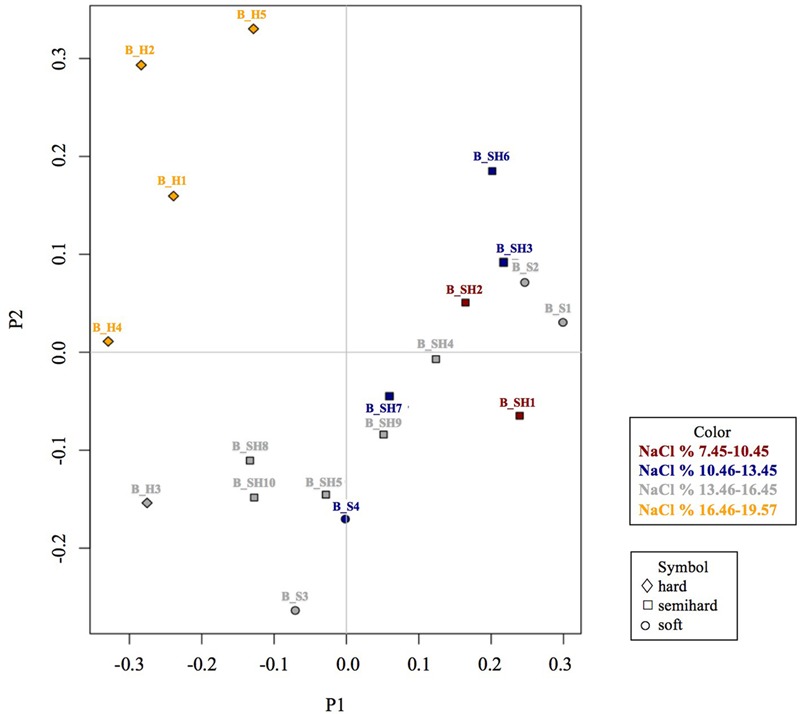
Principal Coordinate Analysis (PCoA) of brine microbiota at the OTU level using Bray-Curtis distances according to cheese type and NaCl level.

Unweighted and weighted Principal Coordinates Analysis (PCoA) highlighted a similar result. In **Figure 3**, the weighted PCoA (the Unweighted PCoA is reported in **Supplementary Figure S1**) showed that brines with the highest salinities, i.e., B_H1, B_H2, and B_H5 and which were also used for hard cheese manufacturing, grouped together. Moreover, brines collected from the same plant, e.g., B_S4 and B_SH7, B_S1 and B_S2, and, B_SH3 and B_SH4, tended to group together except for B_SH5 which did not group with B_SH3 and B_SH4 despite being collected from the same plant.

**FIGURE 4:**
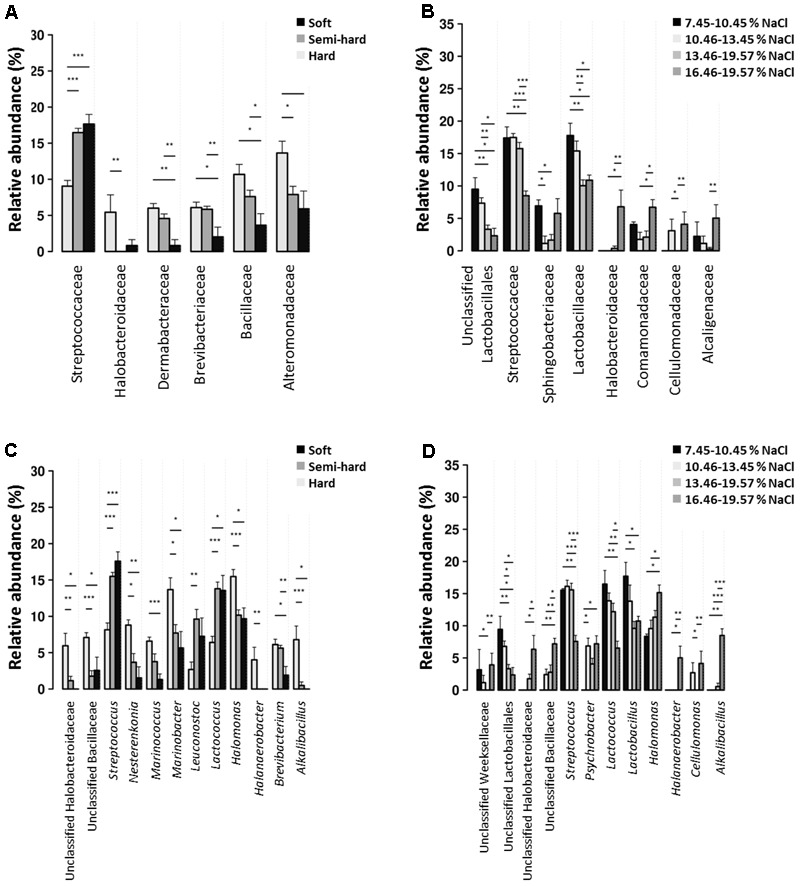
Prevalence of bacterial families **(A,B)** and genera **(C,D)** with significant differences among cheese types **(A,C)** and brine salinities **(B,D).**

The relative abundance of several bacterial families and genera significantly varied according to cheese type (soft, semi-hard, and hard) and salinity (classified in four discrete levels between 7.45 and 19.57%, *p* < 0.05, see **Figure 4**). Members of the *Streptococcaceae* family including *Lactococcus* and *Streptococcus* spp. were significantly more prevalent in brines used for soft and semi-hard cheese manufacturing, while their relative abundance was significantly lower in hard cheese brines (*p* < 0.001). On the contrary, *Halobacteroidaceae* including *Halanaerobacter* spp., *Dermabacteraceae, Brevibacteriaceae* including *Brevibacterium* spp., *Bacillaceae* including *Marinococcus* spp., and *Alteromonadaceae* including *Marinobacter* spp. were more prevalent in hard cheese brines. *Streptococcaceae* (*Streptococcus* and *Lactococcus*) and *Lactobacillaceae* (*Lactobacillus* genus) were more abundant at low salinities while the contrary was observed for *Halobacteroidaceae* (*Halanaerobacter* genus), which were more frequent at the highest salinities. The presence of *Sphingobacteriaceae, Comamonadaceae*, and *Alcaligenaceae* was the highest at the lowest salinities and decreased gradually as NaCl percentage increased. However, at the highest salinity (>16.45%), the frequency of these families was similar to that at the lowest salinities.

## Discussion

In this research, nineteen commercial brines used to salt Italian cheeses were characterized for physicochemical parameters, microbiological counts and handling procedures at the cheese plant. Brine handling, that was manual in most cases, reflects artisanal cheesemaking practices widely used in Italian cheese plants (Maifreni et al., 2013a). However, for most hard cheese associated brines, handling of brines was automatic, which can avoid the formation of concentration gradients within the same brine, thus ensuring homogeneity of the brining process. For Italian hard cheeses, the brining step strongly contributes to syneresis, which helps reducing the water content thus ensuring a very long shelf-life (Malacarne et al., 2008). Moreover, to improve the salt penetration within the curd, hard cheeses like Grana Padano or Parmigiano Reggiano are soaked for long time period (up to 20 days). Soft and semi-hard cheeses are instead soaked in brine for a short period (ranging from 40 min to 1 h for soft cheeses, and for 24 to 72 h for semi-hard ones), because they have a high water content, which facilitates and accelerates the osmotic exchange processes with the brine. Probably because increasing temperature increases salt diffusion rate, temperature of brines for hard cheeses had the highest temperatures, brines used for soft and semi-hard cheeses were instead stored at lower temperatures. For these cheese varieties, a slower penetration rate of salt is required, which is obtained by maintaining brines at low temperatures. Moreover, soaking curds into cool brines could help preventing cheese defects, especially early blowing (Alichanidis, 2007).

Large NaCl concentrations were due to inter-factory cheesemaking procedures, curd dimensions, salting time, as well as variations in milk composition and curd composition prior to salting. The highest values were observed in brines used for hard cheeses, which was expected given the product type. Concentrations lower than 10 g/100 g observed in two brines are considered as hazardous for the appearance of “slimy” defects on the cheese surface (Guinee, 2007c). The lowest pH values detected within soft cheese brines are justified by the fact that soft cheeses undergo an acid-rennet coagulation and usually enter in brines at lower pH than those of cheeses with a coagulation predominantly performed by rennet, as done for hard and semi-hard cheeses. Moreover, the weak structure of soft cheeses allows organic acids, mainly lactic acid, to get out of the curd more rapidly than in semi-hard and hard cheeses. Nevertheless, the highest acidity values were not found in brines for soft cheeses. As a matter of fact, acidity is related not only to the presence of organic acids but also to protein acidic groups, whose presence in the brines tends to gradually increase over time. Acidity is affected not only by the type of soaked cheese but also by the use duration and daily workload. For this reason, acidity is considered a chemical brine aging index (Guinee, 2007a). It should be noted that excessive brine acidity may lead to protein precipitation and a high loss of water at the curd surface, which in turn reduces salt uptake (Guinee and Fox, 2004). Total protein showed large intra-variety differences. Noteworthy, similar contents were found in previous studies (Innocente et al., 2009; Marino et al., 2015).

Microbial counts in brines depend mainly on their handling at the cheese plant and salt concentration (Temelli et al., 2006); however, our results suggested that other factors might be involved, since high total viable counts were detected both in brines with low and high salinities. Brines are usually rich in nutrients coming from the curd, which make them an excellent nutritive medium for halophilic and/or halotolerant microorganisms (Bintsis et al., 2000). As an example, lactic acid bacteria, reached in most cases concentrations higher than 3 log CFU/mL. Microstaphylococci and yeasts counts were mainly higher than 4 and 3 log CFU/mL, respectively. Such concentrations are probably warranted by the ability of these microorganisms to withstand the environmental stresses (e.g., acidic pH, high salt concentrations, and low temperature) that characterized cheese brines. Their role in brines is quite controversial, since they can contribute to the formation of the cheese sensory characteristics; however, by metabolizing lactic acid, free amino acids and compounds derived from protein degradation these microorganisms may be responsible for a pH increase, thus improving the chance of survival for pathogenic and spoilage bacteria (Bintsis et al., 2000). The occasional presence – in soft and semi-hard cheeses – of coliforms, which are sensitive to salt, may suggest a recent contamination coming from the curd, especially in the case for which raw milk was processed, and might be related to early blowing defects in cheese (Quiberoni et al., 2008). As for *S. aureus*, its presence was probably linked to the ability of this microbial species to withstand high salt concentrations (Marino et al., 2011). The presence of potentially pathogenic microorganisms in cheese brines may represent a risk of cross-contamination between products, and certainly, represents a major alarm that signals the need to proceed with rapid brine regeneration or replacement.

Since high salinities may be associated with the induction of a VBNC state in microbes (Pinto et al., 2011), in this study the microbiological data were integrated with high-throughput 16S rRNA gene amplicon sequencing to define the diversity and abundance of microbial communities in cheese brines. Diversity indices exhibited a high bacterial heterogeneity within all brine samples, and interestingly, the highest diversity was associated with the highest salt concentration. This evidence is rather unexpected, as one might think that high salinity has a strong selective effect on the microflora. Our results clearly showed that salt concentration may not be the only factor that determines microbial biodiversity in cheese brines.

*Firmicutes, Proteobacteria, Actinobacteria*, and *Bacteroidetes* were prevalent in brine communities, in accordance with the *phyla* commonly detected in the dairy environment (Riquelme et al., 2015; Sattin et al., 2016). Interestingly, candidate divisions GN02 and TM7 were occasionally found in brines. The GN02 candidate division was first described in the Guerrero Negro hypersaline microbial mat (Ley et al., 2006). Members of this group were recently identified within mammal oral microbiomes (Camanocha and Dewhirst, 2014), whereas this is the first time that GN02 taxa are reported within food environments. As for the TM7 candidate division, it is a commonly encountered bacterial lineage with no cultured representatives, whose members have been identified in soil, fresh groundwater, seawater, and mammalian clinical samples (Hugenholtz et al., 2001). In this study, the TM7 candidate division was detected in two brines used for hard cheese manufacturing and, to our knowledge, their presence is also reported for the first time in the food environment.

Members of *phylum Euryarcheota* were detected in seven out of nineteen brines in spite of specificity of primers for *Bacteria*. The source of members of *Euryarcheota* might be the salt used to prepare the brines, in which they can withstand the high salt stress. In a recent study, it was observed that food-grade salt can harbor up to 10^5^ CFU/g of *Archaea* (Henriet et al., 2014). In spite of the presence of *Archaea* in salt used to prepare cheese brines, they have never been detected on cheese rinds, probably due to the fact that they fail to grow in this environment being outcompeted by other microbial groups including bacteria and fungi. On the contrary, *Archaea* belonged to the non-dominant microbial population found in the core of a Mexican ripened cheese (Escobar-Zepeda et al., 2016).

The most abundant families were *Lactobacillaceae, Moraxellaceae, Halomonadaceae, Streptococcaceae, Micro-coccaceae, Pseudomonadaceae* and *Staphylococcaceae*, indicating that the main microbial sources of cheese brines are soaked curds and salt, as these families are quite widespread within those environments (Carraro et al., 2011; Unno et al., 2015). The presence of *Bifidobacteriaceae* in 15 brines is quite unexpected since the members of this family usually cannot grow at NaCl concentrations higher than 5% (De Castro-Cislaghi et al., 2012). Probably, they correspond to strains adapted to high salinities, which therefore could make them valuable probiotic candidates for application in foods with high salt content such as cheeses or fermented sausages. Some of the families detected in brines are psychrophilic, and their prevalence could be attributed mainly to the low temperatures at which they are stored. As usual before microbiological analyses, the brine samples were transported to the laboratory at +4°C. Due to logistical reasons, the samples arrived at the laboratory within 4 h, except for three samples, which arrived within 12 h. It might be conceivable that lowering the temperature could have favored the prevalence of psychrophilic flora. However, we assumed that a time interval not exceeding 12 h could not allow this type of microflora to grow at significant levels. Indeed, according to data reported on scientific literature and databases (Baranyi and Tamplin, 2004) the environmental conditions of the brines (pH, NaCl concentration, T) seemed not to allow a significant microbial growth of almost all of the microbial genera that make up the core microbiome of used brines within the first 12 h. Thus, it could be considered that such values could have caused almost negligible variations in the prevalence of microbial species adapted to low temperatures.

Nine genera made up the core microbiome of cheese brines. The presence of *Streptococcus, Lactobacillus*, and *Lactococcus* can certainly be traced back to the milk, selected starters or adjunct cultures used for cheesemaking as well as surfaces and equipment. These genera prevailed in brines used to salt soft and semi-hard cheeses, which are characterized by the lowest salinities. These OTUs may belong to starter LAB which have limited salt-tolerance, while *Lactobacillaceae* found at highest NaCl concentrations may be non-starter lactic acid bacteria (NSLAB), which are adventitious lactic acid bacteria that contaminate cheeses and are known for their tolerance to salt and acid stress (Crow et al., 2001; Morales et al., 2011). It has been recently highlighted that a selection of mesophilic lactobacilli and enterococci, which are quite halotolerant, was likely to occur in brines (Esteban-Torres et al., 2014). *Halobacteroidaceae* including *Halanaerobacter* spp., *Brevibacteriaceae* including *Brevibacterium* spp., *Bacillaceae* including *Alkalibacillus* and *Marinococcus* spp., *Alteromonadaceae* including *Marinobacter*, members of the *Dermabacteraceae* family as well as *Halomonas* spp. were instead more abundant in hard cheese brines, which are characterized by high salinities. Such families and genera comprise sporeformers and/or organisms commonly found in marine environments, which make salt their likely source. Considering their marine origin, they are likely well adapted to withstand stresses of cold and hypersaline environments what brines are (Li et al., 2013; Undabarrena et al., 2016). The presence of *Sphingobacteriaceae, Comamonadaceae*, and *Alcaligenaceae* was the highest at the lowest salinities and decreased gradually as NaCl percentage increased. However, at the highest salinity, the frequency of these families was similar to that at the lowest salinities, thus, salinity is probably not the factor driving variations in this microorganisms’ abundance.

*Tetragenococcus* spp. and *Staphylococcus* spp. probably owe their prevalence to their halotolerant nature. To our knowledge, this is the first report of genus *Tetragenococcus* in the dairy environment of Italian cheeses, while it was recently detected in a Polish cheese (Alegría et al., 2012). *Tetragenococcus* might be of relevance for cheese ripening since some species have proven to be proteolytic and lipolytic, thus contributing to sensorial properties of ripened products (Morales et al., 2011). *Staphylococcus* spp. can also contribute to the ripening process thanks to their proteolytic and lipolytic activities, but some of them can also represent a safety issue due to their ability to harbor transferable antibiotic resistance trait, hemolytic activity, amino-acid decarboxylase activities and enterotoxin production (Bartolomeoli et al., 2009). Indeed, *S. aureus* was occasionally detected in brines using a selective culture media, probably coming from mastitic or sub-mastitic milk and the dairy environment. This pathogen has a great relevance in the dairy industry since it frequently causes foodborne outbreaks due to the presence of enterotoxins released in the product. Also, coagulase-negative staphylococci were frequently detected. Some of them, like *Staphylococcus epidermidis* and *S. sciuri*, can form biofilms on environmental surfaces, which could lead to an increase and spread of pathogenic strains (Marino et al., 2011). *S. equorum* was widespread within brines. This species is recognized as a species traditionally associated with cheese (Coton et al., 2010), and has an important role in the flavor in smear ripened cheeses as well as color as it produced carotenoid pigments (Mounier et al., 2006b). Interestingly, very recently, an *S. equorum* strain isolated from cheese brine showed a very strong antibacterial activity against *Listeria monocytogenes* on cheese surfaces (Bockelmann et al., 2017). In the present study, *S. equorum* was detected in all analyzed brines, suggesting that cheese brines could be a source of bacterial strains that could control the spread of *L. monocytogenes* in the dairy environment. It is well documented that, in spite of modern hygiene concepts and improved technology, contamination with this microbial pathogen still occurs sporadically. After a significant increasing trend after 2008, the number of human listeriosis cases stabilized in 2015; however, the presence of this pathogen in the food chain still remains a big threat, particularly in some age groups (EFSA, 2016).

*Halomonas*, a genus already observed in cheese (Coton et al., 2012), is likely to come from salt as it is naturally found in salterns and seawater (Lim et al., 2004; Oren, 2008), whereas *Acinetobacter* probably originates from milk or environmental sources, and it is occasionally found on cheese rinds, suggesting that brines might act as a reservoir of such bacteria in the dairy environment (Masoud et al., 2011). It should be highlighted that *Acinetobacter johnsonii*, which was detected in almost all samples, is now recognized as an increasingly important opportunistic pathogen causing a wide spectrum of nosocomial infection (Akbulut et al., 2014). The genus *Idiomarina*, which was present in all brines, comprises marine deep-sea bacteria that contaminate cheese brines most likely through salt, and to date, it has never been detected in the dairy industry, and only once in fish products (Riddle et al., 2013). Some strains belonging to this genus have been shown to produce lipases with good stability in extreme conditions, which can offer significant contributions and are suitable for harsh industrial processes, such as the production of biodiesel from vegetable oils (Li et al., 2014). Despite their poor tolerance to salt, different species of *Pseudomonas* were found in brines. They probably come from raw milk used for cheesemaking and may survive in brines due to their psychrotrophic nature. In fact, brines are usually stored at low temperatures which, despite not being a selective condition, may help them persist in such a harsh environment. Their presence in the dairy environment is strictly connected to spoilage; moreover, they are frequently found as resident microbiota on surfaces, where they can protect pathogens from sanitation treatments or desiccation (Andreani et al., 2014; Maifreni et al., 2015).

Brines were occasionally found to be contaminated by bacteria of potential hygienic relevance, such as *Bacillus, Clostridium*, and *Neisseria*, as well as members of *Enterobacteriaceae*. Most of them likely originate from the milk used for cheesemaking. These bacteria may be potential spoilers that cause, for example, cheese late blowing (e.g., *Clostridium* spp.) or can form biogenic amines under favorable conditions (e.g., coliforms) (Maifreni et al., 2013b). In this study, coliform viable counts were low, so their presence does not seem to be a real issue. However, brines repeatedly used might act as a reservoir for such bacteria in the dairy environment. Furthermore, opportunistic pathogens were found in repeatedly used brines, such as *Propionibacterium acnes*, which causes skin wounds, and *Shewanella algae*. Regardless of the pathogenic or opportunistic microorganism, it should be emphasized that contaminated brine can, in turn, contaminate the cheese surface and core, which is particularly problematic for those cheeses with an edible rind or no rind, such as Mozzarella cheese. In this case, it is necessary to implement immediate actions for brine sanitization and to identify the possible contamination sources.

Several species with a technological role as well as a probiotic potential were frequently detected. *Corynebacterium variabile*, present in most samples, is part of the surface microbiota on smear cheeses and plays a role in cheese flavor and textural properties, and the same can be said for *Microbacterium maritypicum* and *Vibrio* spp., that were, however, only occasionally present (Schroder et al., 2011). *Pediococcus acidilactici* and *Staphylococcus equorum* are instead recognized as potential producers of bacteriocins (Martino et al., 2013). In this context, used brines might be a source of candidate functional strains too. Indeed various lactobacilli, *Leuconostoc mesenteroides* and *Tetragenococcus halophilus*, are known for their probiotic properties (Masuda et al., 2008; Diana et al., 2015; Innocente et al., 2016; Marino et al., 2017). Given the high salt concentrations in brines, it is conceivable that strains present in such environment would be quite resistant to osmotic stress. In an industrial environment, osmotic pressure represents one of the major stresses encountered by probiotic bacteria during yogurt and cheese production and ripening, meat fermentation, as well as of fish and soy sauce fermentations. Moreover, the formulation and preservation of these cultures impose specific constraints, including the osmotic stress occurring during freeze-drying. It is well known that osmotic stress is a prominent pressure that can produce a decrease in growth rate or survival and affect metabolic activities (Sunny-Roberts and Knorr, 2008). Considering the high prevalence of potentially probiotic species in cheese brines, they may constitute a source of new potential candidate probiotics for application in salted or ripened foods.

The comparison of bacterial brine communities was performed using a multivariate permutation Adonis test, which showed that, among all the technological and physicochemical factors considered, only cheese type, cheese plant, and salinity significantly shaped β-diversity. Indeed, in some cases brines collected from the same plant tended to group together in a PCoA analysis, and the same for brines with the highest salinities, which were also used for hard cheese manufacturing. In spite of the small number of samples belonging to each category, these results would suggest that the main drivers of brine microbiota diversity are the set of interventions that the product encounters on its journey from milk to cheese curd (e.g., milk type, cooking temperature, acidification kinetics), all acting as potential vectors and/or selectors for microbes, as well as the processing environment that may serve as an important reservoir for a facility-specific microbiome. Moreover, within the different brine physicochemical characteristics, only salinity seemed to be accountable for differences in microbial diversity, whereas pH, acidity and protein content did not. These latter parameters are more related to the extensive use of brines over long periods and their levels change throughout their use.

## Conclusion

Results from the present study clearly showed a wide bacterial diversity among cheesemaking brines. The main parameters affecting bacterial diversity were the cheese type, brine salinity, and cheese plant while other physicochemical factors related to brine aging did not seem to impact microbial diversity. Used brines could be a potential reservoir of pathogenic species, e.g., *S. aureus*, thus the frequency of regeneration processes should be carefully taken into account. On the other hand, brines may also be a reservoir of technologically relevant taxa which might be employed to improve technological and functional properties of dairy and non-dairy foods.

## Author Contributions

MM: conception and design of the work, carrying out of culture-dependent analyses, analysis and interpretation of the data, drafting and revising the work. NI: design of the work, carrying out of physicochemical analyses, analysis and interpretation of the data, drafting and revising the work. MiM: carrying out of culture-dependent analyses, analysis and interpretation of data, revising the work. JM, JC-D, and EC: statistical analyses and interpretation of the metagenomic data, drafting and revising the work. BC: carrying out of culture-independent analyses, analysis and interpretation of the data, drafting and revising the work. LC: carrying out of culture-independent analyses, drafting and revising the work.

## Conflict of Interest Statement

The authors declare that the research was conducted in the absence of any commercial or financial relationships that could be construed as a potential conflict of interest.

## Supplementary Material

FIGURE S1 Principal Coordinate Analysis (PCoA) of brine microbiota at the OTU level using Jaccard distance according to cheese type and NaCl level.
