# Supplementary figures and images for: Diversity within Italian Cheesemaking Brine-Associated Bacterial Communities Evidenced by Massive Parallel 16S rRNA Gene Tag Sequencing
Source: Front Microbiol. 2017 Nov 3;8:2119. doi: 10.3389/fmicb.2017.02119 (PMC5675859; doi:10.3389/fmicb.2017.02119)

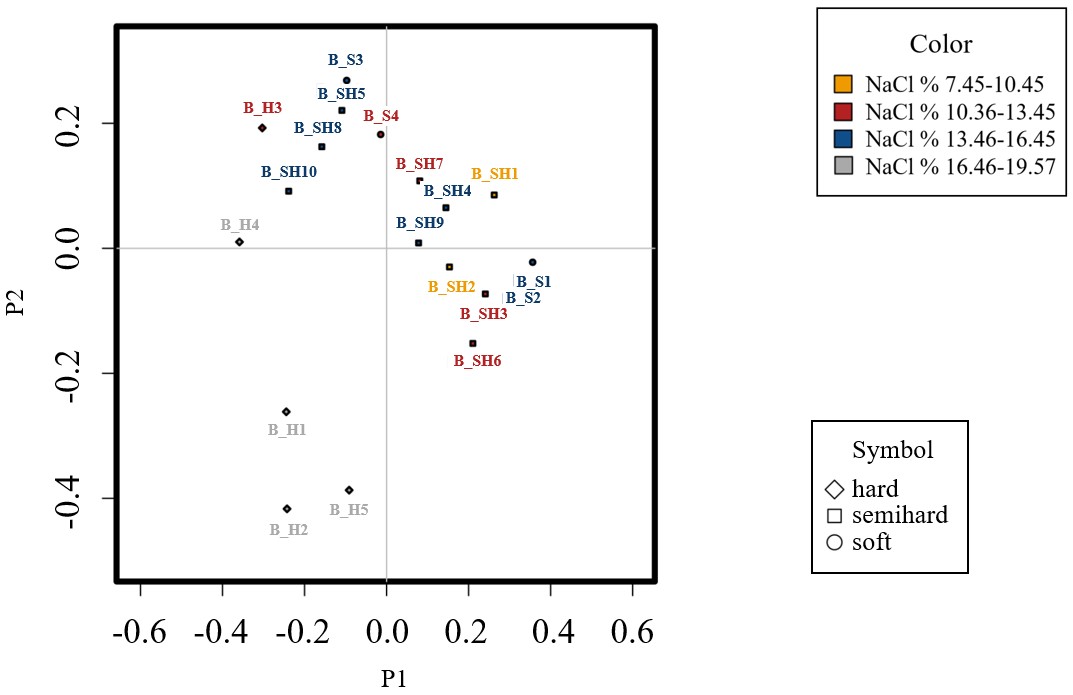

Supplement: FIGURE S1 — Principal Coordinate Analysis (PCoA) of brine microbiota at the OTU level using Jaccard distance according to cheese type and NaCl level. [file Image_1.JPEG]
