# Supplementary material for: Diversity within Italian Cheesemaking Brine-Associated Bacterial Communities Evidenced by Massive Parallel 16S rRNA Gene Tag Sequencing
Source: Front Microbiol. 2017 Nov 3;8:2119. doi: 10.3389/fmicb.2017.02119 (PMC5675859; doi:10.3389/fmicb.2017.02119)
Supplement: Supplementary file 2 [file Table_1.DOCX]

**Table 1S**. Information on cheese type and handling methods associated with the brines

| Brine | Plant code | Cheese variety | Temperature (°C) | Soaking time | Tank capacity | Daily load^*^ | Curd handling | Use^§^ |
| --- | --- | --- | --- | --- | --- | --- | --- | --- |
| B_S1 | A | Soft | 11 | 1 h | 600 L | 50 kg | manual | 135 d |
| B_S2 | A | Soft | 11 | 90 min | 1,200 L | 130 kg | manual | 3 weeks |
| B_S3 | B | Soft | 11 | 40 min | 1,000 L | 90 kg | manual | 6 months |
| B_S4 | F | Soft | 10 | 1 h | 1,200 L | 150 kg | automatic | 9 months |
| B_SH1 | B | semihard | 12 | 48 h | 3,000 L | 450 kg | manual | 11 months |
| B_SH2 | C | semihard | 11 | 24 h | 1,000 L | 120 kg | manual | 12 months |
| B_SH3 | D | semihard | 12 | 48 h | 400 L | 43 kg | manual | 9 months |
| B_SH4 | D | semihard | 11 | 48 h | 900 L | 100 kg | manual | 2 months |
| B_SH5 | D | semihard | 10 | 24 h | 500 L | 55 kg | manual | 2 months |
| B_SH6 | E | semihard | 8 | 48 h | 1,500 L | 190 kg | manual | 7 months |
| B_SH7 | F | semihard | 12 | 24 h | 4,500 L | 350 kg | automatic | 1 week |
| B_SH8 | N | semihard | 12 | 24 h | 1,300 L | 130 kg | manual | 7 months |
| B_SH9 | O | semihard | 13 | 22 h | 1,000 L | 130 kg | manual | 2 months |
| B_SH10 | P | semihard | 12 | 18 h | 1,100 L | 150 kg | manual | 2 weeks |
| B_H1 | G | Hard | 16 | 25 d | 30,000 L | 230 kg | automatic | 6 months |
| B_H2 | H | Hard | 15 | 18 d | 60,000 L | 310 kg | automatic | 10 months |
| B_H3 | I | Hard | 15 | 20 d | 13,000 L | 90 kg | automatic | 8 months |
| B_H4 | L | Hard | 16 | 18 d | 28,000 L | 190 kg | automatic | 3 months |
| B_H5 | M | Hard | 14 | 27 d | 13,000 L | 85 kg | automatic | 12 months |

^*^, values estimated considering the mean weight of curd before salting

### ^§^, values expressed as the time elapsed since the last regeneration or replacement
