# Supplementary material for: Diversity within Italian Cheesemaking Brine-Associated Bacterial Communities Evidenced by Massive Parallel 16S rRNA Gene Tag Sequencing
Source: Front Microbiol. 2017 Nov 3;8:2119. doi: 10.3389/fmicb.2017.02119 (PMC5675859; doi:10.3389/fmicb.2017.02119)
Supplement: Supplementary file 3 [file Table_2.docx]

**Table 2S**. OTUs (number of reads) identified to the species level in brines

| Species | B_S1 | B_S2 | B_S3 | B_S4 | B_SH1 | B_SH2 | B_SH3 | B_SH4 | B_SH5 | B_SH6 | B_SH7 | B_SH8 | B_SH9 | B_SH10 | B_H1 | B_H2 | B_H3 | B_H4 | B_H5 |
| --- | --- | --- | --- | --- | --- | --- | --- | --- | --- | --- | --- | --- | --- | --- | --- | --- | --- | --- | --- |
| *Acinetobacter johnsonii* | 493 | 4464 | 36 | 102 | 170 | 6 | 21 | 2 | 1 |  | 27 | 8 | 748 | 3 | 62 | 2 | 2 | 1 | 27 |
| *Agrococcus jenensis* |  |  |  |  |  |  |  |  |  |  |  |  |  |  | 2 | 17 |  | 2 |  |
| *Anoxybacillus kestanbolensis* |  |  | 1 |  |  |  |  |  |  |  | 6 |  | 4 | 135 | 13308 | 9 | 1 |  | 5 |
| *Brevundimonas diminuta* | 4 |  |  |  |  | 2 |  | 1 |  | 1 |  |  |  |  |  | 24 |  |  | 11 |
| *Corynebacterium variabile* | 60 | 220 | 2 | 8 | 1 | 18 | 12 | 4 |  | 49 | 4 |  |  |  | 26 | 614 | 1 | 7 | 6251 |
| *Haererehalobacter salaria* |  |  | 93 | 136 |  | 1 | 3 | 36 | 548 | 9 | 5 | 6 | 25 |  | 1 | 1 | 69 | 6 | 22 |
| *Halanaerobacter lacunarum* |  |  |  |  |  |  |  |  |  |  |  |  |  |  |  | 9 |  |  |  |
| *Janthinobacterium lividum* |  |  |  |  |  | 3 |  |  |  |  |  |  | 1 |  |  |  |  |  | 4 |
| *Kocuria rhizophila* | 2 | 10 |  | 5 |  |  |  | 9 |  | 21 | 46 | 1 | 3 | 1 | 15 | 26 |  |  | 1439 |
| *Lactobacillus brevis* |  |  |  |  |  | 3 |  | 5 | 1 | 32 |  |  |  |  | 9 | 164 |  | 2 | 64 |
| *Lactobacillus reuteri* |  |  |  |  |  |  |  |  |  |  |  |  |  |  |  |  |  |  | 9 |
| *Lactobacillus zeae* | 6 | 5 |  |  | 1 | 940 | 668 | 613 | 31 | 308 | 1 |  | 2 |  |  | 10 |  | 1 | 18 |
| *Leuconostoc mesenteroides* | 47 | 253 | 5 |  | 125 | 8 | 20 | 123 | 13 | 174 | 1 | 1 | 5 |  | 1 |  |  | 2 | 7 |
| *Marinobacter hydrocarbonoclasticus* |  |  |  |  |  |  |  |  |  |  |  |  | 1 | 46 |  |  |  |  |  |
| *Marinomonas primoryensis* |  |  |  | 1 |  |  |  |  |  |  |  |  |  |  | 12 |  |  |  |  |
| *Microbacterium maritypicum* |  | 1 |  |  |  |  |  |  |  |  |  |  |  |  |  |  |  |  | 9 |
| *Microbispora rosea* |  |  |  |  |  |  |  |  |  |  |  |  |  |  |  | 6 |  |  | 11 |
| *Pediococcus acidilactici* |  |  |  | 1 |  |  |  |  |  | 1 | 2 |  |  |  |  |  |  |  | 7 |
| *Planococcus maitriensis* |  |  |  | 1 |  |  |  |  |  |  |  |  |  |  | 13 |  |  |  |  |
| *Propionibacterium acnes* |  |  |  | 1 |  |  | 1 |  |  |  | 1 | 1 |  | 4 |  | 13 |  | 1 | 10 |
| *Pseudoclavibacter bifida* | 3 | 7 |  |  |  | 16 |  |  |  |  |  |  |  |  | 2 | 10 |  |  | 21 |
| *Pseudomonas alcaligenes* |  |  |  |  |  |  |  |  |  |  |  |  |  |  |  | 16 |  |  | 2 |
| *Pseudomonas balearica* |  |  |  |  |  |  |  |  |  |  |  |  |  |  | 26 | 3 |  |  |  |
| *Pseudomonas stutzeri* |  |  |  |  |  |  |  |  |  |  |  |  |  |  | 35 | 45 |  |  |  |
| *Pseudomonas veronii* | 1 | 3 | 2 |  | 1 |  | 1 | 1 |  |  |  |  | 2 |  | 1 | 6 |  |  |  |
| *Pseudomonas viridiflava* | 1 | 2 |  | 1 |  |  | 1 |  |  | 2 |  |  | 3 |  |  |  |  |  | 51 |
| *Psychrobacter marincola* |  |  |  |  |  |  |  |  |  |  |  |  | 3 |  | 25 | 18 |  |  |  |
| *Salinivibrio costicola* | 1 |  | 2824 | 41 | 2 | 15 | 1 | 10 | 124 |  | 40 | 6 | 1 | 8396 |  | 5 |  | 1 |  |
| *Shewanella algae* | 1 | 2 | 1 | 9 | 1 | 7 | 3 | 2 |  | 2 | 6 | 14 | 8 | 53 | 15 | 1533 | 6 | 3 | 119 |
| *Sphingobacterium faecium* |  | 4 |  |  |  | 2 |  |  |  |  |  |  |  |  |  |  |  |  |  |
| *Sphingobacterium mizutaii* |  |  |  |  |  | 9 |  |  |  |  |  |  | 1 |  | 3 | 45 |  |  |  |
| *Sphingobacterium multivorum* | 2 | 9 |  |  | 3 | 6 |  |  |  |  |  |  |  |  | 1 | 60 |  |  | 1674 |
| *Staphylococcus aureus* | 4 | 1 |  | 35 | 1 |  | 144 | 47 | 12 | 1 | 23 | 37 |  |  |  | 1 | 2399 | 2 | 5 |
| *Staphylococcus epidermidis* |  |  |  |  |  |  | 1 |  |  |  |  |  | 1 | 10 |  |  |  |  | 37 |
| *Staphylococcus equorum* | 5 | 36 | 4 | 222 | 14 | 18 | 13451 | 1452 | 298 | 15 | 69 | 133 | 10 | 27 | 82 | 114 | 47896 | 22 | 548 |
| *Staphylococcus sciuri* |  |  | 1 | 1 |  |  | 2 | 2 |  |  |  | 1 |  | 4 | 1 | 40 | 3 |  | 47 |
| *Staphylococcus succinus* |  | 1 |  |  |  |  |  |  |  |  |  |  |  |  |  |  |  |  | 5 |
| *Stenotrophomonas acidaminiphila* |  | 1 |  |  |  |  |  |  |  |  |  |  | 2 |  |  | 64 |  |  | 1 |
| *Tetragenococcus halophilus* | 10121 | 226 | 40431 | 12260 | 66556 | 6 | 1152 | 2228 | 1535 | 6169 | 35740 | 41515 | 1402 | 4 | 17 | 210 | 15195 | 65 | 57 |
